# Commentary: Adequate 25(OH)D moderates the relationship between dietary inflammatory potential and cardiovascular health risk during the second trimester of pregnancy

**DOI:** 10.3389/fnut.2022.1042324

**Published:** 2022-11-02

**Authors:** Li Zhang, Tianjiao Zhang, Chuanbao Zhang

**Affiliations:** ^1^National Center for Clinical Laboratories, Institute of Geriatric Medicine, Beijing Hospital/National Center of Gerontology, Beijing Engineering Research Center of Laboratory Medicine, Chinese Academy of Medical Sciences, Beijing, China; ^2^Department of Biochemistry, National Center for Clinical Laboratories, Peking Union Medical College, Chinese Academy of Medical Sciences, Beijing, China

**Keywords:** 25-hydroxyvitamin D, pregnancy, liquid chromatography tandem mass spectrometry, immunoassays, vitamin D binding protein

## Introduction

The commentary aims to provide a constructive critique on the chosen method for 25(OH)D measurement in the important and innovative study (1). As described, one of the objectives of this study is to investigate if vitamin D status modifies the association of pro-inflammatory diets with the cardiovascular risk among pregnant women. To assess vitamin D nutritional status for pregnant women at 16–23 gestational weeks, 25(OH)D concentrations in their blood samples were determined by commercial chemiluminescence immunoassay kits (DiaSorin Stillwater, MN, United States). The problem with the chosen 25(OH)D immunoassay is that, changes in sample matrix associated with pregnancy can have a significant impact on the accuracy of 25(OH)D immunoassays, including the DiaSorin assay used in this study (2–5). Inaccurate measurement results may have negative impact on the generation of study results.

## Evidence and interpretation

Circulating 25(OH)D is measured commonly using liquid chromatography tandem mass spectrometry (LC-MS/MS) or immunoassays (2). As 25(OH)D is transported by vitamin D binding protein (VDBP) in bloodstream, it needs to be dissociated from VDBP before testing (2, 6). Unlike LC-MS/MS methods that use strong chemical solvents to extract 25(OH)D, immunoassays have been speculated to fail to release 25(OH)D completely when the concentrations of VDBP increase (5). During pregnancy, VDBP concentrations are increased by estrogen (2, 5). As a result, incomplete dissociation of 25(OH)D by immunoassays may occur, resulting in the under-recovery of 25(OH)D. As shown in Heijboer's (5) study, there was an inverse relationship between VDBP concentrations and deviations of 25(OH)D concentrations from LC-MS/MS results for immunoassays, including Diasorin assay. Cavalier et al. (4) showed that the DiaSorin assay underestimated 25(OH)D levels in pregnant women considerably. Specifically, the mean bias was ~ −44% for the DiaSorin assay, when serum samples from pregnant women were tested. The large negative bias means that a pregnant woman with 25(OH)D levels of 50 nmol/L may have a measured result of 28 nmol/L by the DiaSorin assay, and she will be misclassified to the group with 25(OH)D < 50 nmol/L. Conceivably, a portion of pregnant women in the study by Yin et al. have been misclassified based on the test results by the Diasorin assay, so in fact, the 25(OH)D < 50 nmol/L group also included pregnant women with 25(OH)D ≥ 50 nmol/L, and the unsuccessful stratification according to 25(OH)D concentrations may have negative impact on the final statistical results.

This study also found that the mean concentration for all pregnant women (16–23 gestational weeks) was about 38 nmol/L, and only 21.6% had adequate vitamin D levels, i.e., 25(OH)D concentrations ≥50 nmol/L (1). However, more than half of pregnant women in this study have a vitamin D supplementation frequency of ≥3 days/week. Since it has been demonstrated that supplementing VD > 3 times/week reduces the risk of Vitamin D deficiency during pregnancy significantly (7), it seems unreasonable that the cohort in this study still have a high prevalence (78.4%) of vitamin D deficiency. Furthermore, the prevalence (78.4%) is much higher than that (33.56%) reported by Shen et al. for the second trimester pregnant women using LC-MS/MS. As both cohorts in studies by Shen et al. and Yin et al. were from southeastern China, we can speculate that it's the immunoassay-related underestimation of 25(OH)D that causes the apparent discrepancy in the prevalence of vitamin D deficiency in pregnancy.

## Discussion

Researchers should pay more attention to the immunoassay-related underestimation of 25(OH)D in samples from pregnant women, as it will lead to unreliable study results and poor comparability of studies. At present, standardized LC-MS/MS method can avoid analytical problems observed in immunoassays and serve as the gold standard for 25(OH)D measurement. Therefore, when conducting studies involving 25(OH)D determination in pregnant women, standardized LC-MS/MS method should be considered as the first choice.

## Author contributions

LZ wrote the manuscript. TZ and CZ were the guarantors of this work and revised the manuscript. All authors read and approved the final manuscript.

## Conflict of interest

The authors declare that the research was conducted in the absence of any commercial or financial relationships that could be construed as a potential conflict of interest.

## Publisher's note

All claims expressed in this article are solely those of the authors and do not necessarily represent those of their affiliated organizations, or those of the publisher, the editors and the reviewers. Any product that may be evaluated in this article, or claim that may be made by its manufacturer, is not guaranteed or endorsed by the publisher.
